# A Randomized Study to Examine the Ability of a Caffeine-Based Energy Drink to Impact Energy Expenditure, Fat Oxidation, and Cognitive Performance

**DOI:** 10.3390/nu17233793

**Published:** 2025-12-03

**Authors:** Joesi Krieger, Alex Schrautemeier, Anthony Hagele, Connor Gaige, Olivia Mennemeyer, Sydney Tolbert, Joshua Iannotti, Chad Kerksick, Chris Noonan, Petey Mumford

**Affiliations:** 1 Exercise and Performance Nutrition Laboratory, College of Science, Technology and Health, Lindenwood University, St. Charles, MO 63301, USA; jmorey@lindenwood.edu (J.K.); aschrautemeier@lindenwood.edu (A.S.); ahagele@lindenwood.edu (A.H.); ojm309@lindenwood.edu (O.M.); skt365@lindenwood.edu (S.T.); ji156@lindenwood.edu (J.I.); 2 Dominantly Inherited Alzheimer Network Trials Unit, Department of Neurology, Washington University School of Medicine, St. Louis, MO 63108, USA; gaige@wustl.edu; 3 Health Guidance, Inc., Santa Monica, CA 90403, USA; chris@healthguidance.us; 4 Department of Biomedical Sciences, College of Osteopathic Medicine, Pacific Northwest University of Health Sciences, Yakima, WA 98901, USA; pmumford@pnwu.edu

**Keywords:** energy drinks, fat oxidation, cognition, thermogenic drink, thermogenesis

## Abstract

**Objectives**: This study evaluated the effects of acute and 28-day supplementation with a caffeine-based energy drink on energy expenditure, fat oxidation, and cognitive performance. **Methods**: In a double-blind, placebo-controlled trial, 33 males and 27 females (27 ± 8 years, 26.7 ± 2.2 m/kg^2^) consumed a caffeinated energy drink (200 mg; CAF) or placebo (PLA) for 28 days. Indirect calorimetry assessed energy expenditure and fat oxidation at 0, 30, 60, 90, and 120 min after ingestion on day 1 and 28. Cognition assessments (Dynavision reaction, Serial Sevens, Trail Making Test A (TMT-A) and B (TMT-B)) were performed at 0, 60, and 120 min. **Results**: On day 1, CAF demonstrated higher energy expenditure vs. PLA at 30 (*p* = 0.011), 60 (*p* = 0.001), 90 (*p* = 0.002), and 120 min (*p* < 0.001). On day 28, expenditure remained higher at 30 (*p* < 0.001), 60 (*p* = 0.019), and 90 min (*p* = 0.003). Comparing day 28 to day 1, CAF maintained greater energy expenditure at baseline (*p* = 0.031) with trends at 30 (*p* = 0.057) and 90 min (*p* = 0.051). Fat oxidation was greater with CAF only on day 1 at 60 (*p* = 0.019), 90 (*p* = 0.006), and 120 min (*p* = 0.012). On day 28, CAF showed more correct Dynavision hits (60, *p* = 0.002; 120, *p* = 0.003) and fewer misses (60, *p* = 0.003; 120, *p* = 0.005) vs. PLA. Faster reaction time occurred in CAF at 120 min on day 1 (*p* = 0.028), while serial subtraction showed trends toward higher counts in CAF (day 1: *p* = 0.079; day 28: *p* = 0.059). On day 28, CAF increased perceived focus and energy at 60 and 120 min (focus: *p* = 0.012, *p* = 0.026; energy: *p* = 0.005, *p* = 0.029). Alternatively, a trend for slower TMT-A performance emerged in CAF at 60 min on day 28 (*p* = 0.075), resulting in PLA having faster times across day 28 vs. day 1 comparisons (*p* = 0.033). **Conclusions**: Acute energy drink consumption enhances energy expenditure, fat oxidation, and some cognitive measures, while 28-day use sustains energy expenditure and select cognitive benefits.

## 1. Introduction

Energy drink consumption has increased rapidly worldwide since the introduction of Red Bull in 1986, with global sales projected to exceed $80 billion by 2025 [1]. These beverages appeal to a wide range of users, from college students to athletes, seeking physical or cognitive enhancement [2,3,4]. Recent analyses of the top 75 commercially available energy drinks show that commercially available energy drinks typically contain numerous ingredients (18.2 ± 5.7 per product), with caffeine present in 100% of formulations at an average dose of 174.4 ± 81.1 mg [2]. Given caffeine’s central role, understanding its contribution to the metabolic and cognitive effects of energy drinks remains a priority.

Caffeine is well-established to influence energy expenditure, substrate metabolism, alertness, and cognitive performance [5]. Upon ingestion, the pharmacokinetic pattern of caffeine is well-established with peak plasma caffeine levels commonly occurring after 60 min with a half-life generally ranging from 4 to 6 h [6,7,8]. Acute caffeine intake increases resting metabolic rate in a dose-dependent manner [9,10] and can enhance attention, vigilance, and reaction time, particularly at low (~40 mg or ~0.5 mg/kg) to moderate (~150–300 mg or ~1.5–3 mg/kg) doses [11,12,13,14,15]. Beyond these effects, caffeine has been shown to enhance neuromuscular coordination and neurocognitive function. A 200 mg dose reduced lane violations during extended driving periods [16], and in a study of 100 healthy adults, the same dose improved driving-simulation performance [17].

Energy drinks often contain additional ingredients such as B-vitamins, herbal extracts, or amino acids [2]. Several B-vitamins possess key roles in energy metabolism [18], yet evidence remains limited regarding whether these ingredients meaningfully augment the metabolic or cognitive effects of caffeine alone [19,20,21]. This uncertainty underscores the importance of examining the combined effects of caffeine and additional ingredients commonly included in multi-ingredient energy drinks. Several studies show that acute ingestion of thermogenic or caffeine-containing drinks increases resting energy expenditure and, in some cases, fat oxidation or subjective energy [22,23,24,25]. Acute cognitive benefits have also been documented; for example, consumption of a commercially available energy drink improved psychomotor vigilance and reaction time in exercise-trained men and women [26], suggesting that, leading the authors to conclude that a single serving may enhance aspects of attention and reaction speed. While these findings demonstrate that single-dose ingestion can elicit measurable metabolic and cognitive responses, much less is known about whether these effects persist, adapt, or accumulate with repeated daily consumption of multi-ingredient, caffeine-based energy drinks.

Only a limited number of studies have evaluated the effects of daily or repeated ingestion of multi-ingredient energy drink formulations. Roberts et al. [23] reported that consuming a caffeine-containing energy drink for 28 days increased resting energy expenditure and circulating free fatty acids and also reduced body fat percentage and fat mass compared to placebo. However, studies have also highlighted that much of these observed effects may be attributable primarily to caffeine rather than auxiliary ingredients. Miles-Chan et al. [27] found that a multi-ingredient, sugar-free energy drink and a caffeine-matched control of water and caffeine produced comparable increases in energy expenditure, indicating a limited contribution from the non-caffeine components. Similarly, Pereira et al. [28] found no differences in mood, attention, handgrip strength, or muscular endurance between an energy drink and a caffeine-matched control in exercise-trained adults. Collectively, these findings indicate that many of the observed effects of energy drinks driven primarily by caffeine, though the potential for additive or synergistic effects from other ingredients has not been fully explored. Further, research examining the metabolic and cognitive consequences of repeated daily consumption remains limited thus underscoring the importance of investigating both the acute and sustained effects of multi-ingredient energy drinks on human physiology and cognition.

Following this approach, the present study aims to investigate the metabolic and cognitive effects of a caffeine-based, multi-ingredient energy drink after a single dose and after 28 days of daily supplementation in healthy, recreationally active men and women. By examining both immediate and potential adaptive effects of regular consumption on resting metabolism and cognitive function, this research seeks to address gaps in the understanding of low-dose caffeine-based energy drink supplementation and its impact on non-exercise metabolic and cognitive outcomes.

## 2. Materials and Methods

### 2.1. Experimental Design

**Figure 1:**
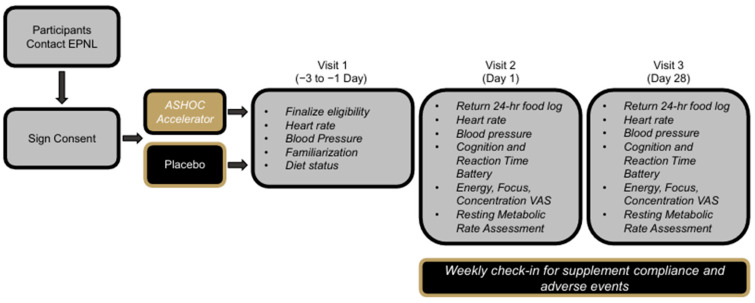
Overview of study design.

This study was conducted using a randomized, double-blind, placebo (PLA)-controlled, parallel group study design in accordance with the Declaration of Helsinki. Three in-person visits were completed at approximately the same time (0600–1000 h) and included screening and familiarization (Visit 1), baseline (Visit 2, Day 1), and end of study (Visit 3, Day 28 ± 3) visits. Prior to each visit, participants were asked to refrain from nicotine, alcohol, and caffeine or energy-drink beverages for 12 h, exercise for 24 h, and food and drinks with calories for eight hours. Compliance was verbally confirmed upon arrival. During Visit 1, all participants signed an IRB-approved consent form (Lindenwood University IRB #: IRB-23-95, approval date: 7 June 2023), provided a health history, and completed a caffeine consumption questionnaire to document baseline habitual caffeine use. During this visit, participants also had their height, weight, body mass index (BMI), resting heart rate and blood pressure assessed to finalize eligibility into the study. Eligible participants were then scheduled for Visit 2 and then instructed on the cognition and reaction time tests and were given an opportunity to practice each of the assessments. Participants were instructed to complete a 24 h food recall the day before Visit 2. This food record was copied and given back to the participant for them to replicate 24 h prior to Visit 3. Upon arrival for Visit 2, participants first had their body mass, hemodynamics, and body temperature measured. Participants then completed a series of visual analog scales (VAS) to evaluate perceptual indicators of affect and cognitive reaction tests before completing their first resting metabolic rate assessment. After completion of all baseline assessments and in a randomized, double-blind, placebo-controlled fashion, participants consumed the first dose of their assigned beverage. The placebo (PLA) and caffeinated energy drink (CAF) were isocaloric, had identical organoleptic properties and were packaged in identical, label-free containers. VAS and cognitive and reaction tests were completed 60 and 120 min after drink ingestion while resting metabolic rate was subsequently evaluated 30, 60, 90, and 120 min. Upon completion of all testing, participants were provided with instructions to consume their assigned beverage daily for 28 consecutive days. Participants returned for visit 3, which was identical to visit 2, whereby the final dose was consumed in the laboratory. An overview of the testing and study design is provided in Figure 1. This study was prospectively registered on 10 August 2023, at Clinicaltrials.gov (NCT05998096).

### 2.2. Study Participants

**Figure 2:**
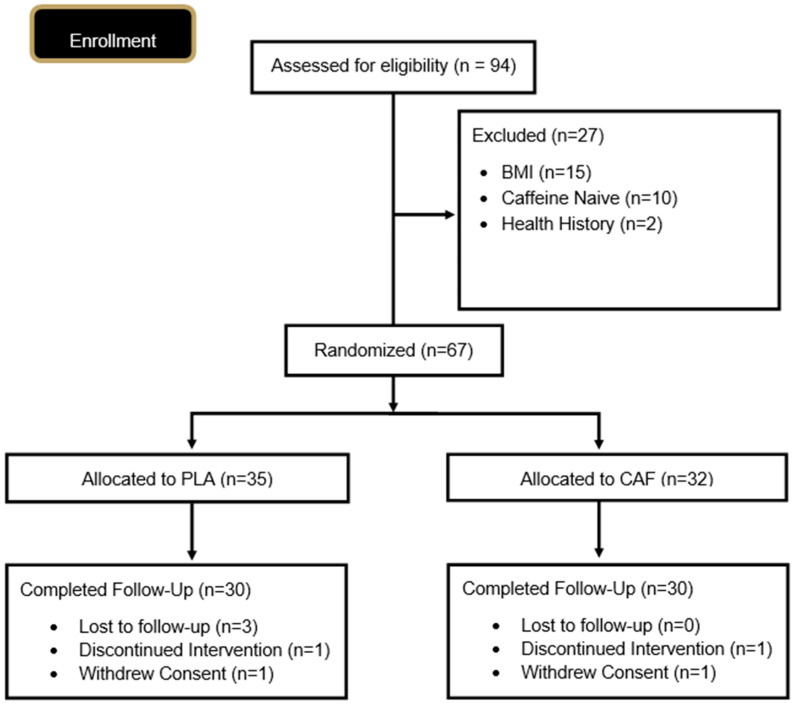
CONSORT diagram.

Sixty healthy adults (33 M, 27 F; 27 ± 8 years, 173.6 ± 10.4 cm, 80.9 ± 12.4 kg body mass, 26.7 ± 2.2 kg/m^2^) who reported consuming an average of 75 mg or more of caffeine per day were recruited to participate in this study (Table 1). The baseline values were descriptively comparable across the CAF and PLA groups. Participant flow through the study is illustrated in the CONSORT diagram (Figure 2). Inclusion criteria for this study were age (30–60 years), healthy and free of disease (as determined by the health history screening questionnaire), a BMI between 24.0 and 31.99 kg/m^2^, physically active (at least 2 days of aerobic and/or resistance training per week over the previous six months), and moderate caffeine users (at least 75 mg per day). Any individual diagnosed with or being treated for any cardiac, respiratory, circulatory, musculoskeletal, metabolic, immune, autoimmune, hematological, neurological, endocrinological disorder or disease, cancer within the past five years, or psychiatric disorder that required hospitalization in the prior 12 months were excluded from the study. Participants were also excluded if they were lactating, pregnant, or planning to become pregnant during the duration of the study, had a history of alcohol or substance abuse in the six months prior, were a current smoker, or presented with an abnormality or obstruction of the gastrointestinal tract precluding swallowing and digestion. Additionally, participants were required to complete a 60-day minimum washout period following consumption of an investigational product or supplement deemed to have a potential influence on any outcome of the study.

### 2.3. Procedures

#### 2.3.1. Anthropometric and Hemodynamic Assessments

Height was measured to the nearest 0.5 cm using a wall-mounted stadiometer (HR-200, Tanita Corp, Inc. Tokyo, Japan) during Visit 1. Body mass was measured bare feet with a t-shirt and athletic shorts to the nearest 0.1 kg at each visit using a level self-calibrating digital balance (Tanita BWB-627A, Tokyo, Japan). Fat and fat-free mass were determined using a bioelectrical impedance analyzer (InBody 570, InBody, Republic of Korea) during Visit 1. Resting heart rate and blood pressure were assessed after five minutes seated rest using an automated sphygmomanometer (Omron BP785, Omron Corporation, Kyoto, Japan).

#### 2.3.2. Dietary and Caffeine Protocol

Habitual caffeine intake from all sources (coffee, tea, soft drinks, energy drinks, food, and supplements) was assessed using a standardized caffeine-consumption questionnaire at baseline. Participants completed a 24 h hand-written food log prior to Visit 2. A copy of this log was returned to participants for replication of diet prior to Visit 3. To determine baseline caloric and macronutrient intake, participants also completed a 24 h recall using the ASA24 online tool (National Cancer Institute, Bethesda, MD, USA) [29]. To further standardize pre-test nutritional status, participants were provided with one identical pre-packaged, frozen meal to consume the evening before both metabolic testing visits. This assisted participants in ensuring caloric and macronutrient intake as well as meal timing remained consistent for the 24 h prior to each testing day. Participants were also instructed to maintain their typical dietary habits throughout the 28-day supplementation period and refrain from intentional changes in caffeine consumption, caloric intake, macronutrient composition, or meal timing.

#### 2.3.3. Cognitive and Reaction Time Battery

Participants were read a standardized instructions script prior to each timepoint, baseline, 60 and 120 min, for all cognitive and reaction time assessments and received 60 s of rest between each assessment. The Trail Making Test, parts A (TMT-A) and B (TMT-B) (INPL Trail Making Test app, Motus Design Group, Victoria, BC, CAN), followed methodology previously published in our laboratory [30] and in alignment with Llinas-Regla et al. [31] during visits 2 and 3 to assess changes in attention, flexibility, and visual search and scanning in response to drink ingestion. These assessments were completed on an electronic tablet (3rd Generation; Apple Inc., Cupertino, CA, USA) in a quiet room without visual or auditory distractions. The tablet was placed vertically on a marked location on the center of the table and participants were instructed to keep their index finger on a standardized blue dot placed laterally to the tablet until the test began. Following the TMT-A and TMT-B, participants completed a two-minute computerized Serial Sevens Subtraction Test [32] which involved the repeated subtraction of seven from a randomly selected three digit starting number using the computer number pad. The assessment was scored based on total number entered correctly.

Reaction time and choice reaction time were assessed using the Dynavision^TM^ D2 Visuomotor device (D2; Dynavision International LLC, Cincinnati, OH, USA) in response to drink ingestion during Visits 2 and 3. This device has been previously used as an assessment tool for visual and motor reaction time, cognition, and motor function [33]. The Dyanvision device is a 4’ × 4’ board that can be raised or lowered relative to the height of the participant and contains five concentric circles comprising 64 total target buttons that can be illuminated along with a central LCD display above the innermost ring. The participants completed a 60 s choice reaction test that required the participant to hit only the red lights that appeared on the board while ignoring the green lights and while maintaining eye contact with the LCD display to verbally announce the continuously changing two-digit number on the screen. The red-light reaction time and total number red lights hit correctly was recorded in addition to the number of green lights incorrectly hit. The height of the board and distance participant stood from the board was standardized relative to the participant. The height was set so that the LCD display was eye level and the participant stood at a distance where their fingertips of each hand were able to reach both sides and top and bottom of the board. This assessment was also completed in a quiet room without visual or auditory distractions.

#### 2.3.4. Visual Analog Scales Protocol

Participants completed electronic (Qualtrics, Provo, UT, USA) VAS that evaluated perceptual responses to PLA or CAF ingestion during Visits 2 and 3 at baseline, 60-, and 120 min post ingestion. Each VAS was constructed similarly with a 100 mm line anchored by ‘Lowest Level’ and ‘Highest Level’ to assess subjective ratings of energy, focus, and concentration using an electronic tablet. In each instance, the participant touched the screen and moved an indicator on the scale until it was positioned at a point they felt represented their rating for that scale.

#### 2.3.5. Resting Metabolic Rate Determination

Resting metabolic rate measurements were conducted 0, 30, 60, 90, and 120 min after supplement ingestion Visits 2 and 3 in a thermoneutral laboratory environment devoid of auditory and visual stimuli. For each assessment, participants rested supine for 15 min under a clear, hard plastic canopy with a transparent plastic drape (TrueOne^®^ 2400 Canopy System, ParvoMedics, Sandy, UT, USA) placed over their head and shoulders. The hood was connected to the metabolic cart to collect all expired gases (oxygen and carbon dioxide). Indirect calorimetry was utilized to analyze expired gases, employing a TrueMax 2400 Metabolic Measurement System (ParvoMedics, Sandy, UT, USA). Rates of energy expenditure and rates of carbohydrate and fat oxidation were calculated according to Weir’s calculations [34]. The metabolic cart underwent daily calibration, ensuring less than 2% of the previous day’s calibration factor.

During the assessment, participants were instructed to remain still, awake, and refrain from talking or using their phones to ensure accurate measurements. The resting metabolic rate and fat oxidation were determined using the final 5 min of the 15 min assessment period, with any outliers (defined as values ±3 standard deviations from the mean) removed from this final segment. Once the resting metabolic rate assessment was completed for each timepoint, participants completed other data collection procedures and were instructed to limit activity and rest quietly prior to their next assessment.

#### 2.3.6. Adverse Events Protocol

Adverse Events (AEs) were systematically collected through spontaneous participant reporting, team interactions, and file reviews. All recorded events were systematically categorized using MedDRA system organ class and graded using Common Terminology Criteria for Adverse Events ([CTCAE] Version 5.0, U.S. Department of Health, and Human Services (published: 27 November 2017)). Data on frequency, severity, duration, and outcome of AEs deemed related or potentially related to study involvement were securely recorded in an electronic database.

### 2.4. Supplementation Protocol

Participants received random assignments of either a 10 kcal non-caffeinated placebo (PLA) beverage or a caffeinated energy drink (CAF) (Accelerator Active Energy, Newport Beach, CA, USA). CAF contained 10 kcal of energy and green coffee bean and green tea extract standardized to 200 mg caffeine. Participants were instructed to consume one drink daily at approximately the same time of day for 28 ± 3 days. If a dose was missed, it could be taken later that day but not made up if it extended into the next day. The initial dose was given in the lab during visit 2, and participants self-administered at home thereafter. Participants were given a two-week supply of their assigned beverage where they were required to return to the laboratory to obtain the remaining supply of their assigned supplementation. This visit was used to evaluate the presence of any adverse events and compliance with the study protocol. In addition, and using an online dairy, compliance to the supplementation protocol and any adverse events were recorded daily and evaluated weekly by research team members. Participants were deemed compliant if their achieved compliance fell between 80 and 120%. At study completion, participants completed a post-blinding questionnaire to assess the efficacy of masking and product blinding. This questionnaire asked whether the participants believed they were assigned the CAF or PLA drink, or if they were unsure of which drink they were assigned.

### 2.5. Statistics

Statistical analyses were conducted using Microsoft Excel and R (Version 4.2.1). Continuous variables are reported as mean ± standard deviation and categorical variables as counts and proportions. Statistical significance was set at *p* ≤ 0.05, with trends noted at *p* ≤ 0.10. The a priori aims were to evaluate (1) acute effects on Day 1, (2) sustained effects on Day 28, and (3) whether these responses were maintained over time (habituation).

To confirm overall time- and group-related changes, mixed factorial repeated-measures ANOVAs were performed separately for Day 1 and Day 28 using raw scores, with Group (CAF, PLA) as the between-subject factor and Time (0, 30, 60, 90, 120 min) as the within-subject factor. Greenhouse–Geisser corrections were applied when needed. Mixed factorial ANOVAs were first used to confirm overall Group × Time dynamics on raw data; subsequent planned Δ-contrasts and AUC/iAUC analyses were conducted to locate physiologically meaningful differences.

Given caffeine’s pharmacodynamic profile and the expectation of discrete peaks rather than uniform effects across time, planned change-from-baseline (Δ) contrasts were used to isolate caffeine’s localized effects at each post-ingestion window (post − baseline). Independent-samples *t*-tests (or Mann–Whitney U tests) compared CAF vs. PLA at 30, 60, 90, and 120 min with a holm adjustment to control for multiple comparisons. Effect sizes were calculated using a cohen’s d (≈0.2 small, 0.5 moderate, 0.8 large) and are reported herein.

To summarize each session, area under the curve (AUC) and incremental AUC (iAUC) were calculated over 0–120 min. AUC reflects total exposure, whereas iAUC (baseline-adjusted) captures the acute excursion above baseline. Habituation was evaluated from within-subject changes in baseline (0 min), iAUC (primary), and raw AUC (supportive) between Day 1 and Day 28.

## 3. Results

Compliance to the supplementation regimen was calculated at 98.9% and 99.2% for CAF and PLA, respectively. The post-blinding questionnaire indicated that 56.7% of participants taking CAF were able to accurately detect their assigned product while 43.3% were either incorrect or unsure. Relatedly, 60.0% of participants taking PLA were able to accurately detect their assigned product while 40.0% were either incorrect or unsure.

### 3.1. Dietary Intake

Participants initially recorded their dietary intake during the 24 h prior to day 1 and then replicated this intake for the subsequent visit on day 28. Energy intake and macronutrient content was computed and is contained in Table 1.

### 3.2. Energy Expenditure and Fat Oxidation

Table 2 contains energy expenditure and rate of fat oxidation data. For energy expenditure, on Day 1, a significant group × time interaction (*p* < 0.001) indicating a differential time-course between groups. Planned Δ-contrasts confirmed significantly greater increases in energy expenditure with CAF at 30 (*p* = 0.011, d = 0.33), 60 (*p* = 0.004, d = 0.41), 90 (*p* = 0.005, d = 0.83), and 120 min (*p* < 0.001, d = 1.06) post-ingestion compared to PLA. CAF also produced a large acute thermogenic response (iAUC; *p* = 0.001, d = 0.96) compared to PLA. On Day 28, a significant Group × Time interaction (*p* = 0.011) again indicated a differential time course between groups. Planned Δ-contrasts showed that CAF remained greater than PLA at 30 (*p* = 0.002, d = 0.44), 60 (*p* = 0.038, d = 0.30), and 90 min (*p* = 0.011, d = 0.37), with a trend at 120 min (*p* = 0.072, d = 0.23). CAF also demonstrated a moderate acute thermogenic response (iAUC; *p* = 0.002, d = 0.41) compared with PLA. Finally, comparisons between Day 28 and Day 1 revealed a small trend for higher baseline REE in CAF at 0 min (*p* = 0.064, d = 0.28). CAF also showed a greater total post-ingestion energy expenditure (AUC; *p* = 0.049, d = 0.31) compared with PLA, indicating higher overall metabolic output after 28-day supplementation. In contrast, the change in incremental AUC (iAUC), reflecting the acute thermogenic rise above baseline was not different between groups, suggesting that while total metabolic output increased, the relative post-dose excursion was maintained, providing no evidence of thermogenic habituation.

Regarding fat oxidation, on Day 1, a significant Group × Time interaction (*p* = 0.005) indicated a differential time course between groups. Planned Δ-contrasts confirmed significantly greater increases in fat oxidation with CAF at 60 min (*p* = 0.039, d = 0.63), 90 min (*p* = 0.022, d = 0.76), and 120 min (*p* = 0.035, d = 0.68) post-ingestion compared with PLA. CAF also produced a moderate acute fat-oxidation response (iAUC; *p* = 0.023, d = 0.68). On Day 28, no significant Group × Time interaction was observed, and the planned Δ-contrasts revealed no differences between groups at any post-ingestion timepoint. Similarly, no acute fat-oxidation response was detected for iAUC. Comparisons between Day 28 and Day 1 showed no change in resting fat oxidation, total post-ingestion fat oxidation (AUC), or incremental AUC (iAUC).

### 3.3. Cognition Tests

Table 3 contains Dynavision Choice Reaction, Serial Sevens, and TMT-A and TMT-B data. For Dyanvision Choice Reaction correct hits, on Day 1, a non-significant Group × Time interaction was observed (*p* = 0.782), and the planned Δ-contrasts revealed no differences between groups at any post-ingestion timepoint. Similarly, no acute response was detected for iAUC. On Day 28, a significant Group × Time interaction was observed (*p* = 0.029), indicating a differential time course between groups. Planned Δ-contrasts showed that CAF was greater than PLA at 60 min (*p* = 0.006, d = 0.39) and 120 min (*p* = 0.006, d = 0.37) post-ingestion. CAF also demonstrated a moderate acute response (iAUC; *p* = 0.003, d = 0.42) compared with PLA. Comparisons between Day 28 and Day 1 showed no change in baseline scores or total post-ingestion correct hits (AUC); however, CAF exhibited a moderately greater iAUC (*p* = 0.006, d = 0.40) relative to PLA.

Regarding Dynavision missed hits during the Choice reaction test, on Day 1, a non-significant Group × Time interaction was observed (*p* = 0.708), and the planned Δ-contrasts revealed no differences between groups at any post-ingestion timepoint. On Day 28, a trend toward a Group × Time interaction was observed (*p* = 0.076), suggesting a potential differential time course between groups. Planned Δ-contrasts indicated that CAF participants made fewer misses than PLA at both 60 min (*p* = 0.009, d = 0.37) and 120 min (*p* = 0.009, d = 0.35) post-ingestion. CAF also demonstrated a moderate acute improvement in performance (iAUC; *p* = 0.004, d = 0.41) compared with PLA. Comparisons between Day 28 and Day 1 showed no change in baseline scores or total post-ingestion misses (AUC); however, CAF exhibited a moderately lower iAUC (*p* = 0.019, d = 0.36) relative to PLA.

Dyanvision average reaction time during the choice reaction test, on Day 1, a trend for the Group × Time interaction was observed (*p* = 0.064), and the planned Δ-contrasts revealed trends that indicated potential faster reaction times in CAF compared to PLA at 60 min (*p* = 0.096, d = 0.50) and 120 min (*p* = 0.096, d = 0.53), and a trend for faster acute improvements in performance (iAUC; *p* = 0.068, d = 0.57). On Day 28, a non-significant Group × Time interaction was observed (*p* = 0.782), and the planned Δ-contrasts revealed no differences between groups at any post-ingestion timepoint. Similarly, no acute response was detected for iAUC. Comparisons between Day 28 and Day 1 showed no change in performance, total post-ingestion performance (AUC), or incremental AUC (iAUC).

Serial Sevens, on Day 1, a non-significant Group × Time interaction was observed (*p* = 0.199), and the planned Δ-contrasts revealed no differences between groups at any post-ingestion timepoint. On Day 28, a non-significant Group × Time interaction was observed (*p* = 0.109), and the planned Δ-contrasts revealed no differences between groups at any post-ingestion timepoint. Comparisons between Day 28 and Day 1 showed no change in performance, total post-ingestion performance (AUC), or incremental AUC (iAUC).

TMT-A completion times, on Day 1, a non-significant Group × Time interaction was observed (*p* = 0.655), and the planned Δ-contrasts revealed no differences between groups at any post-ingestion timepoint. On Day 28, a non-significant Group × Time interaction was observed (*p* = 0.188), and the planned Δ-contrasts revealed no differences between groups at any post-ingestion timepoint. Comparisons between Day 28 and Day 1 showed no change in baseline performance or iAUC. A small trend was observed for total post-ingestion performance (AUC; *p* = 0.089, d = 0.284), potentially, indicating that caffeine may influence processing speed or task efficiency at either visit.

TMT-A errors, on Day 1, a non-significant Group × Time interaction was observed (*p* = 0.608), and the planned Δ-contrasts revealed no differences between groups at any post-ingestion timepoint. On Day 28, a non-significant Group × Time interaction was observed (*p* = 0.195), and the planned Δ-contrasts revealed no differences between groups at any post-ingestion timepoint. Comparisons between Day 28 and Day 1 showed no change in performance, total post-ingestion performance (AUC), or incremental AUC (iAUC).

TMT-B completion times, on Day 1, a non-significant Group × Time interaction was observed (*p* = 0.855), and the planned Δ-contrasts revealed no differences between groups at any post-ingestion timepoint. On Day 28, a non-significant Group × Time interaction was observed (*p* = 0.344), and the planned Δ-contrasts revealed no differences between groups at any post-ingestion timepoint. Comparisons between Day 28 and Day 1 showed no change in performance, total post-ingestion performance (AUC), or incremental AUC (iAUC).

TMT-B errors, on Day 1, a non-significant Group × Time interaction was observed (*p* = 0.944), and the planned Δ-contrasts revealed no differences between groups at any post-ingestion timepoint. On Day 28, a non-significant Group × Time interaction was observed (*p* = 0.829), and the planned Δ-contrasts revealed no differences between groups at any post-ingestion timepoint. Comparisons between Day 28 and Day 1 showed no change in performance, total post-ingestion performance (AUC), or incremental AUC (iAUC).

### 3.4. Visual Analog Scales

Table 4 contains visual analog scores for perceived indications of energy, fatigue, focus, and concentration. Energy, on Day 1, a non-significant Group × Time interaction was observed (*p* = 0.398), and the planned Δ-contrasts revealed no differences between groups at any post-ingestion timepoint. On Day 28, a significant Group × Time interaction was observed (*p* = 0.003), indicating a differential time course between groups. Planned Δ-contrasts revealed that CAF participants reported moderately higher energy at 60 min (*p* = 0.029, d = 0.77) and 120 min (*p* = 0.029, d = 0.59) compared with PLA. CAF also produced a moderate acute energy response (iAUC; *p* = 0.001, d = 0.75) compared to PLA. Comparisons between Day 28 and Day 1 showed no change in baseline ratings, total post-ingestion energy (AUC), or incremental AUC (iAUC), suggesting that while caffeine acutely increased perceived energy after 28 days of supplementation, this effect did not further augment over time.

Fatigue, on Day 1, a non-significant Group × Time interaction was observed (*p* = 0.511), and the planned Δ-contrasts revealed no differences between groups at any post-ingestion timepoint. On Day 28, a non-significant Group × Time interaction was observed (*p* = 0.887), and the planned Δ-contrasts revealed no differences between groups at any post-ingestion timepoint. Comparisons between Day 28 and Day 1 showed no change in fatigue, total post-ingestion fatigue (AUC), or incremental AUC (iAUC).

Focus, on Day 1, a non-significant Group × Time interaction was observed (*p* = 0.703), and the planned Δ-contrasts revealed no differences between groups at any post-ingestion timepoint. On Day 28, a significant Group × Time interaction was observed (*p* = 0.007), indicating a differential time course between groups. Planned Δ-contrasts revealed that CAF participants reported moderately higher focus at 60 min (*p* = 0.021, d = 0.69) and 120 min (*p* = 0.027, d = 0.60) compared with PLA. CAF also produced a moderate acute focus response (iAUC; *p* = 0.019, d = 0.70) relative to PLA. Comparisons between Day 28 and Day 1 showed no change in baseline ratings, total post-ingestion focus (AUC), or incremental AUC (iAUC), suggesting that while caffeine acutely increased perceived focus after 28 days of daily supplementation, this effect did not further augment over time.

Concentration, on Day 1, a non-significant Group × Time interaction was observed (*p* = 0.524), and the planned Δ-contrasts revealed no differences between groups at any post-ingestion timepoint. On Day 28, a trend for the Group × Time interaction was observed (*p* = 0.097), indicating a potential differential time course between groups. Planned Δ-contrasts revealed that CAF participants trended towards a small higher concentration at 60 min (*p* = 0.093, d = 0.22) and 120 min (*p* = 0.065, d = 0.28) compared with PLA. However, no differences were observed for acute concentration response (iAUC). Comparisons between Day 28 and Day 1 showed no change in baseline ratings, total post-ingestion focus (AUC), or incremental AUC (iAUC), suggesting that while caffeine acutely increased perceived concentration after 28 days of supplementation, this effect did not further augment over time.

### 3.5. Body Composition and Hemodynamics

No significant differences (*p* > 0.05) in body mass were observed between CAF and PLA at screening, Day 1 (−10.0, 5.4 kg; *p* = 0.569), or Day 28 (−8.63, 4.45 kg; *p* = 0.525), or with the observed changes in body mass throughout the entire study protocol between the two drink conditions (−0.80, 0.60; *p* = 0.871). Further, there were no statistically significant changes in heart rate, systolic blood pressure, or diastolic blood pressure on Day 1, Day 28, or comparisons between Day 28 and Day 1 showed no change in baseline ratings, total post-ingestion focus (AUC), or incremental AUC (iAUC) (Table 5).

### 3.6. Adverse Events

Seven AEs deemed potentially related to the study products are presented in Table 6. Two CAF participants reported four events, including one participant with three separate instances of gastrointestinal symptoms and the other participant reporting agitation. The remaining three AEs were reported by one PLA participant, with an instance of headache, gastrointestinal symptoms, and lethargy occurring concurrently. All AEs were deemed mild in severity. Additionally, and as noted above, no statistically significant changes were observed for heart rate or blood pressure changes.

## 4. Discussion

The aim of this study was to examine the effects of daily ingestion of a caffeine-based energy drink on acute and 28-day changes in energy expenditure, fat oxidation, and cognitive performance in healthy adults. While several studies have evaluated acute metabolic and cognitive responses to caffeine or multi-ingredient energy drinks [22,24,25,26,27,28,35,36], many of these studies have focused on single-dose effects rather than repeated daily ingestion. Given the diversity of commercially available formulations, and the lack of repeated dosing evidence, our study extends the existing literature by evaluating both acute and 28-day responses to daily consumption of a commercially relevant, caffeine-based multi-ingredient energy drink.

The ability of caffeine-containing beverages to acutely increase thermogenesis and resting energy expenditure have been previously reported [10,22,25,27]. The present findings align with this acute pattern but extend the literature by showing a trend toward higher baseline resting energy expenditure on Day 28. Notably, the lack of difference in incremental AUC between Day 28 and Day 1 indicates that the acute, dose-dependent thermogenic response to CAF was maintained over time. Thus, while total metabolic output increased across the 28-day supplementation period, these findings do not support a blunting of the drink’s immediate thermogenic effect, suggesting that habituation to the thermogenic properties of the drink may not occur within this timeframe. In contrast to the sustained thermogenic effects, changes in fat oxidation were limited to the acute response observed on Day 1. These results align with literature demonstrating caffeine’s acute lipolytic effects [10,22], as CAF increased fat oxidation only after the first dose, with no differences between groups at any timepoint on Day 28 and no changes in baseline fat oxidation or total incremental AUC across days. Although mechanistic conclusions cannot be drawn from these data, the disappearance of the fat-oxidation response by Day 28 suggests a potential attenuation of caffeine’s lipolytic effects with daily use. These findings contrast with **Roberts et al.** [23], who reported continued increases in free fatty acids and resting energy expenditure as well as significant reductions in percent body fat following 28 days of energy drink ingestion. While **Roberts et al.** [23] utilized an identical supplementation duration and a similarly aged mixed-sex cohort, the formulation used included taurine, differing amounts of B vitamins, EGCG, yet the same caloric and caffeine content as the present drink. Differences between studies may reflect unrecorded behavioral changes or differences in energy drink formulations. As weight loss remains a common goal for many individuals, future research should explore the mechanisms associated with the increased energy expenditure in the absence of sustained changes in fat oxidation and evaluate the independent and synergistic contributions of non-caffeine ingredients in multi-ingredient energy drinks.

Cognitive outcomes revealed selective improvements. The choice reaction time task demonstrated significant enhancements after 28 days of daily ingestion, suggesting that repeated consumption may be required to elicit measurable cognitive benefits, a finding not commonly reported in prior caffeine and energy drink studies [5,37,38]. However, these improvements emerged only in correct and missed hits on Day 28, with no significant effects observed after the first dose on Day 1 and no corresponding changes in reaction time. Although most Day 28 to Day 1 comparisons did not reveal changes in raw performance, the iAUC analyses for Dynavision correct hits and misses demonstrated significant differences between CAF and PLA across days. This indicates that the acute effects of the drink on the Dynavision task was greater on Day 28 than on Day 1, even though baseline values and total AUC did not change. Thus, the enhanced Day 28 performance appears to reflect an increased acute effect relative to PLA rather than a true longitudinal improvement in underlying cognitive ability. Interestingly, no acute improvements in this task were observed after the first dose, which contrasts with several studies reporting immediate cognitive benefits following single-drink ingestion [11,26,36,39,40]. Many of those studies consisted of similar aged mixed-sex cohorts and used products containing similar caffeine doses (150–200 mg) but different ingredient profiles, such as caffeine alone, or added taurine, glucuronolactone, or higher sugar content. These compositional differences as well as differences in reaction time assessment methods and baseline habitual caffeine intake (ranging from caffeine-naïve to moderate users), as this was the only study to use the Dynavision to record this variable, may partly explain the lack of acute effects. Outcomes from the serial subtraction did not demonstrate significant group differences on either day. Conversely, the trail making test showed trends favoring PLA on day 28, potentially reflecting domain-specific cognitive demands, testing fatigue, or individual variability in responsiveness. Collectively, these findings suggest that cognitive responses to multi-ingredient, caffeine-based energy drinks in this study were domain specific, limited in magnitude, potentially sensitive to habitual caffeine use, and influenced by formulation differences beyond caffeine alone.

Finally, and beyond the changes observed for cognition, participants consuming CAF reported significant improvements in perceived energy and focus 60 min after ingestion on day 28. These results align with prior work evaluating acute perceptual responses to energy drinks [25,36] and are consistent with the well-characterized pharmacokinetics of caffeine [7,8]. In contrast, limited work is available for comparison across multi-week supplementation periods as Roberts et al. [23] and Lockwood et al. [41] did not examine perceptual changes, and Seidler et al. [42] reported positive changes in mood but not the perceptual markers examined here.

The strengths of this study include high reported compliance (>98%), a randomized, double-blind, placebo-controlled design, and the recruitment of healthy adults who regularly consumed caffeine (>70 mg/day). Including regular caffeine consumers may help to mitigate potential confounding effects from caffeine-naive individuals and provide a clearer assessment of habituation [43]. Additionally, the mixed-sex cohort of healthy, recreationally active adults reflects typical energy drink users [44,45]. Participants also replicated their diet 24 h before each visit to reduce variability, and the study maintained external validity by not intervening in daily dietary or caffeine habits beyond asking participants to observe an overnight fast prior to each study visit and requesting maintenance of current routines. Although ample literature exists on acute caffeine intake and growing evidence supports the effects of single-dose energy drinks, fewer data are available on outcomes extending beyond one dose. Future work should examine supplementation beyond 28 days in caffeine-naïve individuals or those that are overweight/obese, as well as the independent and synergistic contributions of non-caffeine ingredients on metabolic and cognitive outcomes. An additional future area of inquiry should explore the efficacy of prolonged energy drink ingestion on cognitive and neuromotor changes in competitive athletes, as improvements in reaction time and decision-making accuracy may meaningfully influence performance.

Key limitations of the present study include the short supplementation period and lack of biospecimen sampling, which limits our ability to directly link the observed metabolic and cognitive outcomes to underlying physiological mechanisms. Although this study extends existing work by incorporating repeated daily dosing across a multi-week period, longer interventions may help clarify whether metabolic or cognitive responses to energy drink ingestion continue to evolve beyond 28 days. Extended supplementation periods may also be necessary to determine whether changes in body composition or training quality emerge, given the well-established performance-enhancing and weight/fat loss effects of caffeine [5]. Finally, we were unable to isolate the impact of individual ingredients in the energy drink formulation used in this study. While prior research strongly suggests caffeine as the primary driver of observed changes [2,28,40], understanding the potential independent and synergistic impact of commonly included ingredients should remain a priority for future studies.

## 5. Conclusions

Collectively, the results of this investigation demonstrate that a caffeine-containing energy drink produces acute increases in energy expenditure and maintains this thermogenic response across 28 days of daily use. Although fat oxidation rose only after the initial dose and did not persist with repeated ingestion, the sustained thermogenic effect suggests limited habituation to the drink’s metabolic properties. Cognitive outcomes were more selective, with significant improvements observed only in Dynavision performance at Day 28 and with these Day 28 effects exceeding the change observed in the placebo group, while most other cognitive tasks showed no meaningful changes. Taken together, these findings indicate that repeated daily consumption of a caffeine-based energy drink may enhance certain aspects of cognitive performance and preserve the acute thermogenic response, though effects are task-specific and not uniformly distributed across cognitive domains.

## Figures and Tables

**Table 1:** Subject demographics.

All Participants (n = 60)	PLA	CAF
	Mean (SD)	Mean (SD)
Age (years)	27 (8)	27 (8)
Weight (kg)	81.6 (13.2)	80.2 (11.6)
Body Mass Index (kg/m^2^)	26.7 (2.2)	26.7 (2.2)
Skeletal Muscle Mass (kg)	34.8 (7.4)	34.9 (7.6)
Bodyfat (%)	25.2 (7.0)	24.0 (7.7)
Females (%)	40.0	51.7
Males (%)	60.0	48.3
Resting Heart Rate (beats/min)	65 (8)	66 (11)
Systolic Blood Pressure (mm Hg)	121 (12)	119 (14)
Diastolic Blood Pressure (mm Hg)	78 (9)	74 (8)
Energy (kcals/day)	2273 (622)	2182 (844)
Carbohydrate Intake (g/day)	220 (97)	223 (126)
Protein Intake (g/day)	124 (60)	109 (51)
Fat Intake (g/day)	101 (35)	97 (38)
Caffeine Intake (mg/day)	248 (157)	252 (122)

Data are presented as means (SD); kg = kilograms; m = meters; mg = milligrams; PLA = placebo; CAF = Caffeinated Energy Drink.

**Table 2:** Energy expenditure and fat oxidation.

Variable			PLA	CAF	
	Visit	Time	Mean ± SD	Mean ± SD	*p* Value
Energy Expenditure (kcals/min)	Day 1	30–0	0.01 ± 0.058	0.074 ± 0.084	0.011 *
		60–0	−0.05 ± 0.095	0.019 ± 0.098	0.004 *
		90–0	−0.029 ± 0.078	0.039 ± 0.086	0.005 *
		120–0	−0.04 ± 0.071	0.047 ± 0.092	<0.001 *
		iAUC	−0.022 ± 0.060	0.039 ± 0.067	0.001 *
	Day 28	30–0	0.012 ± 0.08	0.081 ± 0.065	0.002 *
		60–0	−0.014 ± 0.099	0.027 ± 0.069	0.038 *
		90–0	−0.017 ± 0.086	0.042 ± 0.061	0.011 *
		120–0	−0.016 ± 0.099	0.026 ± 0.094	0.072
		iAUC	−0.007 ± 0.070	0.041 ± 0.044	0.002 *
	D28–D1	0	−0.008 ± 0.129	0.05 ± 0.12	0.064 *
		AUC	0.008 ± 0.099	0.051 ± 0.116	0.049 *
		iAUC	0.016 ± 0.063	0.001 ± 0.056	0.352
Rate of Fat Oxidation (g/min)	Day 1	30–0	−0.013 ± 0.02	−0.005 ± 0.028	0.232
		60–0	0.004 ± 0.027	0.021 ± 0.027	0.039 *
		90–0	−0.003 ± 0.026	0.017 ± 0.027	0.022 *
		120–0	0.002 ± 0.028	0.022 ± 0.028	0.035 *
		iAUC	−0.003 ± 0.019	0.011 ± 0.021	0.024 *
	Day 28	30–0	−0.006 ± 0.022	−0.008 ± 0.022	0.656
		60–0	0.014 ± 0.023	0.02 ± 0.019	0.337
		90–0	0.011 ± 0.025	0.016 ± 0.024	0.656
		120–0	0.014 ± 0.023	0.025 ± 0.017	0.209
		iAUC	0.007 ± 0.018	0.010 ± 0.015	0.162
	D28–D1	0	−0.011 ± 0.054	0.004 ± 0.041	0.479
		AUC	−0.001 ± 0.042	0.003 ± 0.037	0.696
		iAUC	0.010 ± 0.020	−0.001 ± 0.024	0.201

Data are presented as means ± SD; kcal = kilocalories; g = gram; min = minute; Reported *p* values are between group *p* values; * = Significant between group change (*p* < 0.05); PLA = placebo; CAF = Caffeinated Energy Drink.

**Table 3:** Cognition.

Variable			PLA	CAF	
	Visit	Time	Mean ± SD	Mean ± SD	*p* Value
Dynavision Choice Reaction Correct Red Hits (count)	Day 1	60–0	0.07 ± 5.35	0.53 ± 4.22	1.000
		120–0	2.87 ± 5.22	2.70 ± 5.01	1.000
		iAUC	90.00 ± 411.64	102.41 ± 332.96	0.899
	Day 28	60–0	0.20 ± 7.13	4.17 ± 4.76	0.006 *
		120–0	2.27 ± 7.40	6.00 ± 6.76	0.006 *
		iAUC	80.00 ± 618.21	425.17 ± 440.18	0.003 *
	D28–D1	0	1.40 ± 7.91	−0.20 ± 5.85	0.265
		AUC	158.00 ± 584.17	289.66 ± 479.01	0.347
		iAUC	−10.00 ± 712.39	322.76 ± 430.48	0.006 *
Dynavision Choice Reaction Red Misses (count)	Day 1	60–0	−0.23 ± 4.45	−0.62 ± 3.05	1.000
		120–0	−2.13 ± 3.89	−1.66 ± 3.64	1.000
		iAUC	−78.00 ± 333.56	−86.90 ± 231.73	0.905
	Day 28	60–0	0.07 ± 6.31	−2.62 ± 3.47	0.009 *
		120–0	−1.53 ± 6.73	−4.10 ± 5.15	0.009 *
		iAUC	−42.00 ± 561.34	−280.34 ± 321.04	0.004 *
	D28–D1	0	−1.17 ± 6.92	0.34 ± 4.51	0.168
		AUC	−104.00 ± 450.09	−152.07 ± 345.13	0.988
		iAUC	36.00 ± 665.08	−193.45 ± 357.72	0.019 *
Dynavision Choice Reaction Time (sec)	Day 1	60–0	0.00 ± 0.04	−0.01 ± 0.03	0.096
		120–0	−0.01 ± 0.04	−0.03 ± 0.04	0.096
		iAUC	−0.04 ± 3.15	−1.68 ± 2.61	0.068
	Day 28	60–0	−0.01 ± 0.03	−0.02 ± 0.04	0.305
		120–0	−0.02 ± 0.04	−0.03 ± 0.04	0.305
		iAUC	−1.29 ± 2.75	−2.34 ± 3.31	0.143
	D28–D1	0	0.01 ± 0.04	−0.01 ± 0.04	0.242
		AUC	−0.61 ± 2.58	−2.07 ± 3.24	0.185
		iAUC	−1.25 ± 4.42	−0.66 ± 3.83	0.587
Serial Subtraction Test (count)	Day 1	60–0	−1.63 ± 13.90	3.87 ± 14.83	0.158
		120–0	−1.43 ± 14.14	4.43 ± 14.95	0.158
		iAUC	−141.00 ± 1160.30	365.00 ± 1180.79	0.199
	Day 28	60–0	−2.53 ± 15.12	6.63 ± 17.55	0.118
		120–0	3.37 ± 12.71	6.80 ± 18.12	0.141
		iAUC	−51.00 ± 1049.75	602.00 ± 1373.94	0.199
	D28–D1	0	−1.67 ± 14.40	1.77 ± 12.74	0.664
		AUC	−110.00 ± 1149.15	449.00 ± 1263.50	0.235
		iAUC	90.00 ± 1389.23	237.00 ± 1504.76	0.696
Trail Making Test—A Time (sec)	Day 1	60–0	−0.57 ± 3.65	−0.14 ± 2.48	1.000
		120–0	−0.86 ± 4.60	−0.16 ± 2.33	1.000
		iAUC	−59.94 ± 336.87	−14.99 ± 197.36	0.699
	Day 28	60–0	−0.23 ± 1.91	0.80 ± 2.49	0.151
		120–0	−0.15 ± 2.42	0.14 ± 2.02	0.614
		iAUC	−18.70 ± 158.73	52.38 ± 188.94	0.240
	D28–D1	0	−1.03 ± 3.59	−0.22 ± 2.32	1.000
		AUC	−82.39 ± 175.10	33.56 ± 183.36	0.089
		iAUC	41.24 ± 366.67	74.43 ± 305.44	1.000
Trail Making Test A—Errors (count)	Day 1	60–0	−0.07 ± 2.16	0.53 ± 2.21	1.000
		120–0	0.37 ± 2.55	0.55 ± 1.24	1.000
		iAUC	7.00 ± 160.63	45.52 ± 139.02	0.794
	Day 28	60–0	0.00 ± 2.08	0.73 ± 1.96	0.456
		120–0	−0.10 ± 1.88	0.67 ± 1.47	0.263
		iAUC	−3.00 ± 168.12	64.00 ± 140.43	0.533
	D28–D1	0	−0.17 ± 1.97	0.17 ± 1.12	0.821
		AUC	−30.00 ± 149.48	40.34 ± 179.69	0.158
		iAUC	−10.00 ± 177.80	23.79 ± 195.64	0.982
Trail Making Test B—Time (sec)	Day 1	60–0	−2.48 ± 7.33	−1.87 ± 6.62	1.000
		120–0	−4.55 ± 7.61	−3.78 ± 6.54	1.000
		iAUC	−277.17 ± 638.19	−225.22 ± 539.77	0.946
	Day 28	60–0	1.66 ± 7.45	−0.64 ± 6.18	0.397
		120–0	−0.81 ± 5.55	−2.37 ± 4.80	0.397
		iAUC	75.51 ± 544.72	−109.26 ± 453.61	0.318
	D28–D1	0	−4.57 ± 7.45	−2.22 ± 5.89	0.543
		AUC	−177.09 ± 503.71	−150.95 ± 475.07	0.838
		iAUC	367.24 ± 988.41	115.96 ± 659.97	0.543
Trail Making Test B—Errors (count)	Day 1	60–0	0.23 ± 4.59	0.00 ± 3.45	1.000
		120–0	−0.21 ± 2.81	−0.27 ± 3.02	1.000
		iAUC	14.48 ± 326.83	−8.00 ± 269.01	0.848
	Day 28	60–0	1.27 ± 4.23	0.73 ± 4.06	0.763
		120–0	0.20 ± 2.96	−0.30 ± 4.04	0.763
		iAUC	82.00 ± 306.34	35.00 ± 336.66	0.848
	D28–D1	0	−0.30 ± 4.26	0.50 ± 3.75	1.000
		AUC	42.41 ± 246.54	103.00 ± 255.13	1.000
		iAUC	75.52 ± 494.94	43.00 ± 505.37	1.000

Data are presented as means ± SD; sec = seconds; Reported *p* values are between group *p* values; * = Significant between group change (*p* < 0.05); PLA = placebo; CAF = Caffeinated Energy Drink.

**Table 4:** Visual analog scales.

Variable			PLA	CAF	
	Visit	Time	Mean ± SD	Mean ± SD	*p* Value
Energy (mm)	Day 1	60–0	2.86 ± 12.95	7.24 ± 14.69	0.474
		120–0	5.66 ± 14.90	9.59 ± 13.26	0.474
		iAUC	336.43 ± 1105.09	722.07 ± 1217.92	0.216
	Day 28	60–0	0.97 ± 17.71	13.07 ± 13.43	0.009 *
		120–0	7.07 ± 14.14	15.62 ± 15.06	0.029 *
		iAUC	270.00 ± 1378.24	1252.76 ± 1232.18	0.011 *
	D28–D1	0	−2.62 ± 24.74	−7.45 ± 17.47	0.801
		AUC	−482.14 ± 1921.70	−363.10 ± 1532.84	0.801
		iAUC	−83.57 ± 1797.88	530.69 ± 1452.01	0.801
Fatigue (mm)	Day 1	60–0	−3.86 ± 15.20	−7.69 ± 19.42	0.480
		120–0	−6.03 ± 16.76	−10.72 ± 19.06	0.480
		iAUC	−397.50 ± 1322.51	−783.10 ± 1662.76	0.636
	Day 28	60–0	−2.27 ± 18.94	−2.31 ± 16.15	1.000
		120–0	−4.23 ± 18.98	−6.00 ± 17.59	1.000
		iAUC	−263.00 ± 1651.94	−318.62 ± 1432.74	0.710
	D28–D1	0	2.45 ± 24.31	−6.24 ± 24.70	0.182
		AUC	538.93 ± 2236.73	−284.48 ± 2617.08	0.975
		iAUC	144.64 ± 2131.18	464.48 ± 2098.37	0.975
Focus (mm)	Day 1	60–0	2.36 ± 12.39	4.34 ± 13.05	0.597
		120–0	3.69 ± 14.89	6.24 ± 14.19	0.597
		iAUC	234.64 ± 1087.51	447.93 ± 1141.83	0.473
	Day 28	60–0	−0.77 ± 13.58	9.41 ± 15.81	0.021 *
		120–0	3.40 ± 10.74	10.52 ± 13.10	0.027 *
		iAUC	56.00 ± 1075.73	880.34 ± 1265.12	0.019 *
	D28–D1	0	0.14 ± 20.18	−7.45 ± 23.14	0.189
		AUC	−272.14 ± 1604.99	−461.38 ± 1533.53	0.651
		iAUC	−182.14 ± 1529.09	423.41 ± 1849.52	0.425
Concentration (mm)	Day 1	60–0	3.00 ± 13.19	6.79 ± 8.34	0.406
		120–0	6.93 ± 15.10	7.31 ± 10.00	0.608
		iAUC	377.14 ± 1169.40	626.90 ± 732.67	0.131
	Day 28	60–0	1.23 ± 14.8	7.93 ± 15.11	0.093
		120–0	4.53 ± 12.58	10.03 ± 13.08	0.065
		iAUC	210.00 ± 1203.36	776.90 ± 1244.58	0.131
	D28–D1	0	−0.83 ± 22.05	−5.14 ± 18.99	1.000
		AUC	−364.29 ± 1609.38	−466.55 ± 1427.10	1.000
		iAUC	−175.71 ± 1578.09	150.00 ± 1506.78	1.000

Data are presented as means ± SD; mm = millimeter; Reported *p* values are between group *p* values; * = Significant between group change (*p* < 0.05); PLA = placebo; CAF = Caffeinated Energy Drink.

**Table 5:** Hemodynamics.

Variable			PLA	CAF	
	Visit	Time	Mean ± SD	Mean ± SD	*p* Value
Heart Rate (beats/min)	Day 1	60–0	−0.97 ± 5.77	−1.90 ± 6.06	1.000
		120–0	−0.74 ± 4.84	−0.30 ± 6.04	1.000
		iAUC	−62.22 ± 431.38	−123.00 ± 495.73	0.623
	Day 28	60–0	−2.87 ± 6.48	−1.47 ± 3.40	0.341
		120–0	−2.40 ± 5.50	−0.40 ± 5.66	0.341
		iAUC	−244.00 ± 514.50	−100.00 ± 312.20	0.393
	D28–D1	0	1.45 ± 7.71	0.43 ± 4.81	1.000
		AUC	−2.22 ± 547.76	75.00 ± 532.75	1.000
		iAUC	−220.00 ± 667.72	23.00 ± 564.48	0.439
Systolic Blood Pressure (mm Hg)	Day 1	60–0	1.48 ± 9.62	3.57 ± 9.18	0.399
		120–0	1.21 ± 9.66	4.77 ± 7.67	0.257
		iAUC	126.43 ± 756.98	357.00 ± 691.75	0.464
	Day 28	60–0	−0.40 ± 6.80	−2.37 ± 18.65	1.000
		120–0	2.33 ± 8.24	3.10 ± 10.94	1.000
		iAUC	46.00 ± 612.12	−49.00 ± 1259.54	0.796
	D28–D1	0	0.66 ± 11.05	1.97 ± 11.35	0.655
		AUC	19.29 ± 741.99	−170.00 ± 1417.57	1.000
		iAUC	−83.57 ± 1022.79	−406.00 ± 1426.09	1.000
Diastolic Blood Pressure (mm Hg)	Day 1	60–0	1.79 ± 5.60	4.60 ± 6.92	0.184
		120–0	2.04 ± 4.41	2.97 ± 6.38	0.519
		iAUC	170.36 ± 404.07	365.00 ± 560.31	0.267
	Day 28	60–0	0.83 ± 4.68	1.10 ± 5.83	0.846
		120–0	0.43 ± 5.01	2.43 ± 6.85	0.447
		iAUC	63.00 ± 380.32	139.00 ± 509.82	0.516
	D28–D1	0	1.79 ± 6.46	2.67 ± 6.01	0.593
		AUC	95.36 ± 584.24	94.00 ± 772.23	1.000
		iAUC	−123.21 ± 680.85	−226.00 ± 608.21	0.674

Data are presented as means ± SD; mm Hg = millimeters of mercury; Reported *p* values are between group *p* values; PLA = placebo; CAF = Caffeinated Energy Drink.

**Table 6:** Adverse events.

Category	PLA	CAF
Participants Reported	1	2
Headache	1	0
Gastrointestinal	1	3
Agitation	0	1
Lethargy	1	0

Data are presented as counts. Note that all adverse events reported in the table above were mild in severity.

## Data Availability

The raw data supporting the conclusions of this article will be made available by the authors on request.

## Abbreviations

The following abbreviations are used in this manuscript:

AE	Adverse Event
BMI	Body Mass Index
CAF	Caffeinated Energy Drink
CTCAE	Common Terminology Criteria for Adverse Events
DV	Daily Value
EGCG	Epigallocatechin Gallate
PLA	Placebo
TMT-A	Trail Making Test—Part A
TMT-B	Trail Making Test—Part B
VAS	Visual Analog Scales
